# Neighbors affect vocal behavior of tropical wrens: a multispeaker density-manipulation experiment

**DOI:** 10.1093/beheco/arae075

**Published:** 2024-09-17

**Authors:** Natalie V Sánchez, Isabel Vargas-Valverde, María José Espejo-Uribe, Daniel J Mennill

**Affiliations:** Department of Integrative Biology, University of Windsor, Windsor, Ontario, N9B3P4, Canada; Instituto Internacional en Conservación y Manejo de Vida Silvestre, ICOMVIS, Universidad Nacional de Costa Rica, Heredia 1350-3000, Costa Rica; Escuela de Biología, Universidad de Costa Rica, San José 11501-2060, Costa Rica; Departamento de Biología, Universidad Nacional de Colombia, Bogotá 111321, Colombia; Department of Integrative Biology, University of Windsor, Windsor, Ontario, N9B3P4, Canada

**Keywords:** birdsong, female song, multispeaker playback, song rate, territory defense, *Thryothorus rufalbus*

## Abstract

For territorial animals, the behavior of conspecific neighbors sets the social context of communication. Despite numerous investigations of vocalizations related to territory defense and mate attraction, the effect of neighbor density on animal vocal behavior has received little attention, particularly in tropical animals and animals where both sexes produce complex acoustic signals. In this study, we used an innovative multispeaker playback experiment to manipulate the apparent density of neighbors in rufous-and-white wrens, *Thryophilus rufalbus*, living in Costa Rica’s tropical dry forest. In this tropical songbird, both males and females defend year-round territories and sing complex, learned songs for territory defense. We recorded the singing behavior of 24 subjects (12 pairs), and then we used an array of 6 loudspeakers to simulate the presence of 6 new territorial neighbors (3 simulated pairs) outside each subject pair’s breeding territory. The stimuli persisted for 3 consecutive days, with both male and female songs broadcast at a natural rate from dawn to dusk. We found that the mean male song rate increased by almost 50% in response to the simulated increase in local density. Females showed less frequent song-type switching rates following the simulated increase in local density, although it was a marginal increase. These findings reveal that male and female songbirds’ vocal behavior varies with the local density of territorial neighbors. We conclude that birds are sensitive to acoustic signals of conspecific density arising from sounds beyond their territory boundaries, and that they use this public information to guide their vocal behavior.

## Teaser

Territorial animals may vary their behavior when the number of neighbors changes. In a playback experiment with tropical rufous-and-white wrens (*Thryophilus rufalbus*), we used multiple loudspeakers to simulate an increase in the number of neighbors outside birds’ territory boundaries. Males increased their song rate following the simulation. Females changed song types less frequently following the simulation. We concluded that birds use acoustic information regarding the number of neighbors heard beyond their territory boundaries.

Interactions between conspecific individuals shape the behavioral ecology and evolution of animal signals and animal communication. For territorial animals, behaviors associated with resource defense have fundamental consequences for survival and reproduction; individuals that effectively defend resources necessary for survival and reproduction will be favored through natural and sexual selection (Emlen and Oring 1977). Territorial defense behaviors are under external pressures imposed by the social environment, and the influence of nearby conspecific animals is expected to play a dominant role in shaping the behavioral ecology of acoustic signals (Kelly et al. 2018). For example, nearby conspecific signalers affect the signaling behavior of animals, including amphibians, primates, and whales (Fristrup et al. 2003; Bastos et al. 2011; Lameira et al. 2022).

Density is an important social factor that affects territorial behavior (Sánchez and Mennill 2024). Higher numbers of nearby conspecific individuals are expected to modify territorial behavior (Naguib 2005; Searcy and Beecher 2009). At higher densities, more frequent territorial interactions may influence an animal’s patrolling behavior and mate-guarding behavior or drive changes in vocal behaviors in favor of more aggressive displays (Tobias and Seddon 2000; Erne and Amrhein 2008). The acoustic displays of songbirds provide a model system for studying signals used in territory defense between conspecific rivals, with a historical focus on the complex learned songs of males in temperate songbirds (Catchpole and Slater 2008). Songbird vocal behaviors often vary during interactions between neighbors (Goodwin and Podos 2014; Reichert et al. 2022), and song rate, in particular, often varies with more frequent interactions between neighbors (Liu 2004). Although the effects of conspecific density on signaling behavior are recognized in studies of temperate songbirds, the behavior of tropical songbirds has been poorly studied (Sánchez and Mennill 2024), in spite of the high biodiversity of the tropics (Stutchbury and Morton 2022). In tropical territorial birds, both males and females sing, often performing duets and defending year-round territories, and yet the influence of variation in density is virtually unstudied in these animals.

Female song is a poorly studied trait (Langmore 1998; Riebel et al. 2019). In many tropical and southern-temperate bird species, females sing, and this is now recognized as an ancestral trait in songbirds (Odom et al. 2014). Commonly, songs in males serve a territory defense function, and emerging evidence suggests that female song also plays a role in territory defense among other functions (Riebel et al. 2019; Austin et al. 2021; Dargis et al. 2021). One recent correlational study described the changes in vocal behaviors in a tropical species where females and males both sing solo songs and perform duets: rufous-and-white wrens, *Thryophilus rufalbus*. Interestingly, through a long-term analysis of variation in vocal behavior, females appear to vary their song-type switching rate when the number of neighboring pairs changed from having 1 neighbor to 2 neighbors, although it was not different across birds with 0, 1, or 3 neighbors (Owen and Mennill 2021). This observation of an effect of density on the female song, combined with the dearth of studies on the effects of conspecific density on the female song and song of tropical birds, motivated us to design a playback experiment that would evaluate the effects of heightened conspecific density on song in tropical birds.

Employing an innovative multispeaker playback design, we experimentally increased the apparent local density of territorial rufous-and-white wrens. We used 3 pairs of loudspeakers to simulate an increase in local density corresponding to 3 pairs of additional conspecific animals, and we recorded the responses of territorial males and territorial females to the neighborhood-level simulation. We hypothesized that territorial males and females would vary their vocal behaviors when the density of neighbors changed due to heightened competition for resources. We predicted that rufous-and-white wrens would show an increase in the rate of independent and duet songs in response to the playback-simulated increase in local density because independent and duet songs are involved in territory defense (Mennill 2006; Mennill and Vehrencamp 2008). We further predicted that rufous-and-white wrens would vary their independent song-type switching behavior given the results of a previous observational study on our focal species (Owen and Mennill 2021). Song-type switching could be an agonistic response and previous work has revealed lower song-type switching levels during simulated territory intrusion (Molles 2006; Deoniziak and Osiejuk 2020); therefore, we predicted low song-type switching in rufous-and-white wrens following a playback manipulation that increases apparent local density, given that more neighbors should be associated with higher levels of territorial intrusion. Our goal was to expand the limited investigations into the effects of social context on animal communication and vocal responses of females. Furthermore, given the increasing pace of deforestation and habitat loss in the tropics (Curtis et al. 2018), we may anticipate continued declines in the density of conspecific animals in remaining populations, and a better understanding of the effects of local density will provide insights into behavioral responses to habitat change that can inform conservation of tropical animals.

## Materials and Methods

### General field methods

We performed this experiment in the world’s largest remaining stand of tropical dry forest located in Área de Conservación Guanacaste, Sector Santa Rosa, Guanacaste, Costa Rica (10°52ʹN, 85°36ʹʹW; Janzen 1988; Gillespie et al. 2000), from late April to early June of 2022 and 2023. This is a seasonal deciduous forest with 2 annual climatic periods: a dry season that typically occurs from December to May, when precipitation is largely absent; and a wet season that typically occurs from May to November, characterized by heavy rainfall. For the last 2 decades, our research team has studied the rufous-and-white wrens in the mature regions of this tropical dry forest (Mennill and Vehrencamp 2008; Woodworth et al. 2018). In rufous-and-white wrens, both males and females sing complex musical songs, including songs that are sung as solos or duets (Mennill and Vehrencamp 2005), and males and females occupy congruent territories throughout the year (Osmun and Mennill 2011). Duets are created when a bird overlaps their partner’s song, or immediately follows their partner’s song, and make up 1% to 5% of vocalizations uttered by rufous-and-white wrens (Mennill and Vehrencamp 2008; Topp and Mennill 2008). Both males and females have vocal repertoires and sing with eventual variety, repeating each song multiple times before switching to a new song type (Harris et al. 2016). The tropical dry forest experiences very warm weather (Campos et al. 2015; Woodworth et al. 2018), and most wren singing behavior and territorial activity occurs in the first part of the morning, tapering off to lower levels at later times of day (Mennill and Vehrencamp 2005).

Following protocols developed in our laboratory, we located rufous-and-white wrens on their territories, captured birds with mist nets, and banded birds with individually distinctive combinations of colored leg bands. At the time of capture, we collected morphological measurements of each bird including wing length, tarsus length, beak length, beak depth, and weight. Previous work shows that males and females can be distinguished based on their morphological features; sex differences were confirmed based on known acoustic differences between the voices of males and females, as well as breeding activities (only females incubate eggs in this species; Mennill and Vehrencamp 2005).

To map rufous-and-white wren territory boundaries, we conducted 2- or 3-h observation sessions over the course of 2 mornings for each territorial pair, following birds as they engaged in natural activities (i.e. without the use of playback). Birds tend to remain in the same territories over long periods of time (Mennill and Vehrencamp 2008; Graham et al. 2017). During these focal observation sessions, we collected recordings of the songs from the focal pair with a digital recorder (Marantz PMD661 MKII; 44,100 Hz sampling rate, 16-bit depth, WAV format) and a shotgun microphone (Sennheiser MKH70). We created maps for each territorial pair by collecting points with a handheld Global Positioning System, including documented positions for each nest, for common singing perches of the territorial male and female, and for foraging observations observed during focal observations. We also noted the relative position of neighboring individuals, based on hearing songs from more distant birds in adjacent habitats. Among the 12 pairs of playback subjects, the focal pair had 0 neighbors in 4 cases, 1 neighboring pair in 6 cases, and 2 neighboring pairs in 2 cases (see Supplementary Material 1).

### Playback design

**Fig. 1.**
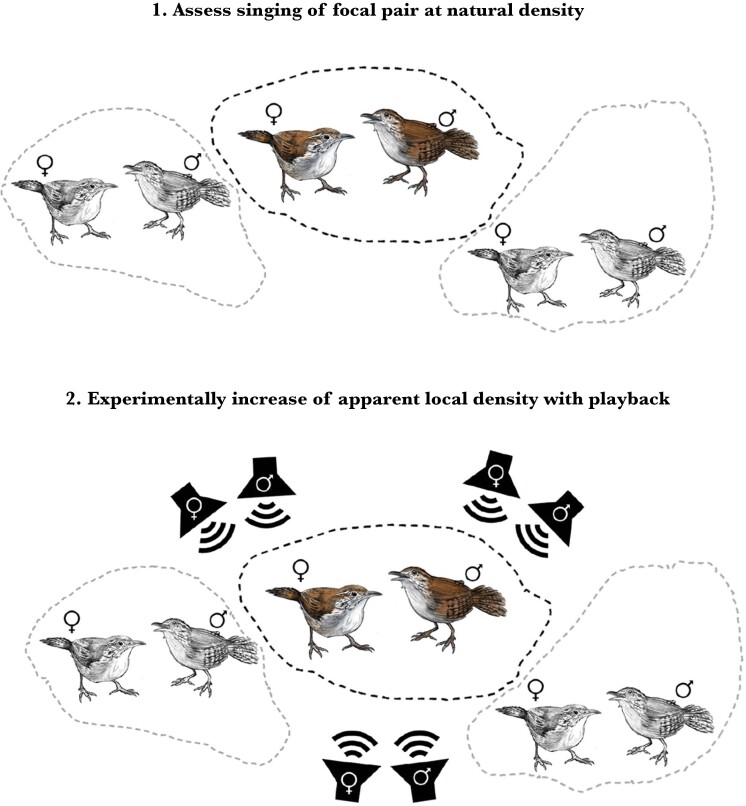
Schematic representation of a playback experiment designed to investigate whether male and female rufous-and-white wrens, *Thryophilus rufalbus*, alter their vocal behavior in response to simulated changes in local conspecific density. Top: A focal pair was selected for playback, their territory boundaries were mapped, and their vocal behavior was recorded. Bottom: For 3 d, a 6-speaker playback apparatus simulated 3 new pairs of territorial birds singing beyond the subjects’ territory boundaries, and the reaction of the focal pair was recorded on the third day of playback. Wren illustrations by Caleb Sequeira Ulate.

We used the territory maps of focal pairs, combined with our detections of neighbors, to select 3 locations outside each playback pair’s territory boundaries where we could experimentally simulate 3 unfamiliar pairs of conspecific neighbors (Fig. 1). For each of the 3 simulated pairs of neighbors, we chose only “empty” areas of habitat where we did not detect any conspecific animals during our focal observations or during other activities during the course of our field research. To ensure that the playback-simulated neighbors were outside the territorial area defended by the playback subjects, we made sure that the speakers were placed at least 90 m from the subjects’ nests, and beyond any area where we had observed the pairs during the preceding focal recordings. We chose 3 areas in 3 different directions from the subjects’ territories, with the speakers oriented toward the focal pair’s territory (Fig. 1). We never performed the experiment on adjacent pairs in close succession, to avoid any familiarity effects that might occur after a neighboring pair had already heard the playback manipulation.

We developed a 6-speaker playback device consisting of 3 stereo pairs of customized digital loudspeakers that had been developed in the course of another experiment on vocal learning in songbirds (Mennill et al. 2018, 2019). Details of the custom-built loudspeakers are provided in Mennill et al. (2018). Each pair of speakers was powered with 8 rechargeable D-cell batteries and a light sensor that turned the speakers on at sunrise and off at sunset. Each pair of loudspeakers was connected with a 16-m cable, allowing us to simulate duets of a male from one loudspeaker, and a female from the other loudspeaker, with a typical distance of separation for this species (Mennill and Vehrencamp 2008). In effect, each of the 3 pairs of loudspeakers was a stereo duet playback design (Douglas and Mennill 2010), each simulating a pair of wrens neighboring the subject, broadcasting songs beyond the subject pair’s territory boundaries. Speakers broadcast the stimuli at an amplitude of 85 dB measured at a distance of 1 m (Scosche SPL1000F sound level meter).

We created playback stimuli using songs recorded from 3 different breeding pairs in our study population in 2011. None of the birds from 2011 were still alive, and therefore these playback stimuli simulated strangers to the playback subjects, although strangers singing population-typical songs (Graham et al. 2018). For each simulated pair of neighbors, we selected 3 male and 3 female songs and pasted the songs into the left and right channels of a stereo sound file using Adobe Audition software. We created the stereo playback tracks to broadcast male and female songs at species-typical rates of male and female songs and typical rates of duetting (following the song and duet rates from unprovoked pairs described in Mennill and Vehrencamp 2005). We created stereo playback tracks with a typical pattern of diel variation, with a dawn chorus followed by lower song rates later in the day (following the song rate data from unprovoked pairs described in Topp and Mennill 2008). Playback began at approximately 0515 h and finished at approximately 1730 h. The first hour of playback stimuli had 120 male songs, 18 female songs, and 10 duets; the second hour had 60 male songs, 12 female songs, and 5 duets; and the third hour had 30 male songs, 6 female songs, and 4 duets. Songs continued at the same rate as the third hour until dusk. The simulated male and female changed song type twice during each hour of playback; we divided each hour into 3 sections of 20 min, and a song-type change from both the simulated male and female initiated each 20-min interval. Each experiment was performed for 4 consecutive days.

### Assessing responses to playback

To assess the responses of territorial pairs of rufous-and-white wrens to the simulation of 3 new pairs of territorial neighbors beyond their territory boundaries, we sampled the singing behavior of birds on the day before the playback experiment began (i.e. day 0), and again on the third morning of the playback experiment (i.e. day 3). We chose to focus our analyses of the birds’ reactions to the playback manipulation on day 3 to ensure that the subjects would have ample opportunity to hear the duets from the simulated birds outside their territory boundaries and to ensure that there was an opportunity for the subjects to assess that these were animals defending territories, rather than prospecting for territories. In year 1 (2022), we recorded 9 focal pairs from May 10 to June 13. In year 2 (2023), we recorded 3 focal pairs from April 25 to May 30. We collected recordings of the subjects’ vocal behavior between 0515 and 0615 h, which corresponds with periods of higher song output for rufous-and-white wrens (i.e. the dawn chorus; Mennill and Vehrencamp 2005). At this time of day, there was only one occasion of rain during day 2 of the experiment; on all other days, the weather was clear or cloudy skies with no wind and no rain, ensuring similar climatic features. We collected these recordings with both in-person focal recordings (equipment described above) and also with automated recorders (Wildlife Acoustics Song Meter Mini; Wildlife Acoustics, Inc., Concord, MA, USA) placed in previously observed singing perches around the subjects’ breeding territory. While collecting recordings, we confirmed that the loudspeakers were positioned outside the territory of the focal pairs’ boundaries by observing that the focal pairs were not singing near the loudspeakers.

In the laboratory, we used sound analysis software (Syrinx-PC; J. Burt, Seattle, WA, USA) to visualize sound spectrograms of the pre-playback period (day 0) and post-playback period (day 3) during a 1-h interval. We analyzed the number of male songs, female songs, and duets. As in previous studies, we defined duets as songs that were overlapping or separated by less than 1.0 s of silence (Mennill and Vehrencamp 2005; Mennill 2006). We created a song library for males and females from recordings collected earlier in the field season, and then differentiated between the songs of the resident male and female by comparing songs to this library; each bird has a unique repertoire (Mennill and Vehrencamp 2005; Harris et al. 2016). We then calculated the following singing behaviors: the number of independent songs sung by the male (i.e. the total number of songs sung by the male as a solo or as the first song in a duet); the number of independent songs sung by the female; the number of duets; and song type switching rate for each sex (i.e. the number of times each bird switched between song types, divided by the number of songs they sang). For duets, in rufous-and-white wrens either sex can create a duet by responding to its partner’s song, but in this dataset, the majority of duets were female-created duets (65.6% on day 0; 73.4% on day 3; mean proportions); therefore, we did not differentiate between female-created and male-created duets, reporting just the total number of duets. For all 5 response variables, we calculated the behavior per hour of recording during both the pre-playback recording (day 0) and post-playback recording (day 3).

### Statistical analyses

For each of the vocal behaviors that we defined as a response variable (independent song rate, duet rate, and song-type switching rate), we used a Linear Mixed Model to test the effect of higher density of conspecific animals on rufous-and-white wren vocal behaviors. We used the R package “lme4” (Bates et al. 2015) to calculate the models. We compared the vocal behaviors before playback (day 0) to the behaviors during playback (day 3) as the independent variable, and we included sex as an independent variable. We included pair identity as a random factor in all models. We reported the Analysis of Variance (ANOVA) output of the coefficients of the model to obtain p-values for the comparisons of the main effects (R package “car”; the output is the Type II Wald Chi-square test). We interpret these models using language of evidence, following Muff et al. (2022), using terms of little or no evidence, weak evidence, moderate evidence, and strong evidence. We also reported the means ± SE for each variable and the effect size ± SE of the models for males and females. All the analyses were conducted in R 4.2.2 (R Core Team).

### Ethical note

This study was conducted with the permission of the Sistema Nacional de Áreas de Conservación of Costa Rica under the research permit number R-SINAC-ACG-PI-026-2022, and the University of Windsor Animal Care Committee (AUPP-20-09). During banding, birds were held for the minimum amount of time possible and released quickly at the site of capture. The observations with binoculars and manual recordings of the songs of the focal subjects were conducted from an adequate distance to avoid disturbing the natural behavior of the birds.

## Results

Following 3 d of 6-speaker playback that simulated the voices of new territorial neighbors beyond the boundaries of territorial pairs of rufous-and-white wrens, birds changed their vocal behavior. Our playback simulated new territorial birds beyond the territorial boundaries of the subjects, not territorial intruders, and our field observations confirm that the subjects did not move into the area where the loudspeakers were located or interact directly with the loudspeakers.

**Fig. 2.**
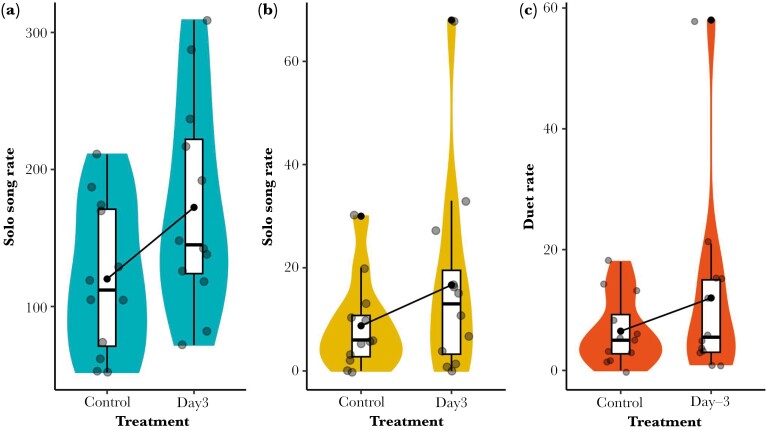
Independent song rate of male (A) and female (B) rufous-and-white wrens, *Thryophilus rufalbus*, before and after a 3-d multispeaker playback treatment simulating higher local density. Independent song rate is expressed as the number of solo songs per hour. (C) Duet rate created by male and female rufous-and-white wrens, *Thryophilus rufalbus*, before and after a 3-d multispeaker playback treatment simulating higher local density. Duet rate is expressed as the number of duets per hour. Boxes show 25th, 50th, and 75th percentiles and whiskers show the lower and upper value within 1.5 times interquartile range below the 25th percentile and above the 75th percentile. Violin plots show the probability density across the full range of data. For each comparison, the means are shown by black dots connected with black lines.

The simulated increase of the number of conspecific neighbors had an effect on male independent song rate, increasing from 120.08 ± 15.88 songs per hour before the playback manipulation (day 0) to 172.33 ± 22.08 songs per hour after 3 d of playback (day 3); this is moderate evidence that male independent song rate increased in response to a playback-simulated increase in local population density (χ^2^_1_ = 5.15, *N* = 12, *P* = 0.02; effect size = 52.20, ± 24.00; Fig. 2A). Although the female independent song rate increased by a factor of 2, from 8.75 ± 2.55 songs per hour before the playback manipulation (day 0) to 16.67 ± 5.55 songs per hour after 3 d of playback (day 3), this is little or no evidence, statistically, that female independent song rate increased in response to a playback-simulated increase in local population density (χ^2^_1_ = 1.83, *N* = 12, *P* = 0.18; effect size = 7.92 ± 6.11; Fig. 2B).

Although the duet rate increased from 6.50 ± 1.63 duets before the playback (day 0) to 12.00 ± 4.57 duets after the playback simulation (day 3), this provides little or no evidence that the duet rate increased in response to a playback-simulated increase in local population density (χ^2^_1_ = 2.22, *N* = 12, *P* = 0.14, effect size = 5.50 ± 3.85) (Fig. 2C).

**Fig. 3.**
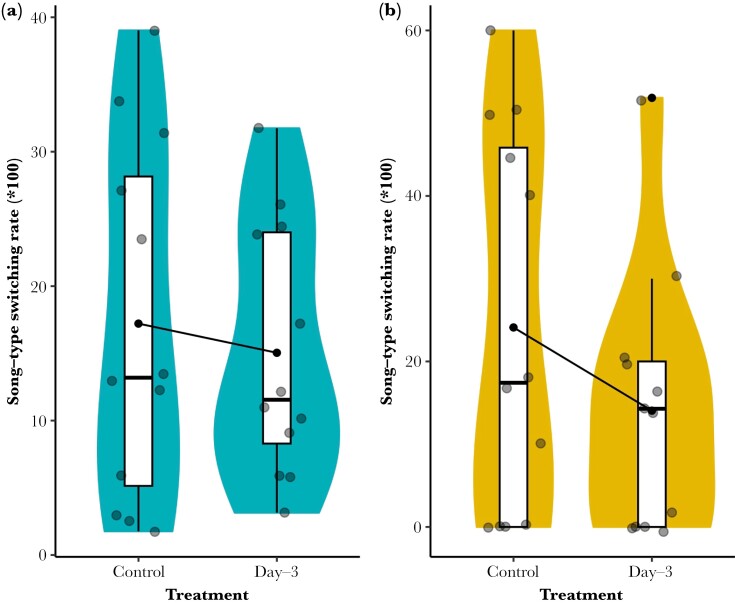
Song-type switching rate (song-type switching/total independent song × 100) of male (A) and female (B) rufous-and-white wrens, *Thryophilus rufalbus*, before and after a 3-d multispeaker playback treatment simulating higher local density. Boxes show 25th, 50th, and 75th percentiles and whiskers show the lower and upper value within 1.5 times interquartile range below the 25th percentile and above the 75th percentile. Violin plots show the probability density across the full range of data. For each comparison, the means are shown by black dots connected with black lines.

Males had a song-type switching rate of 17.21 ± 3.80 changes per hour before the playback (day 0) and 15.04 ± 2.70 changes per hour on day 3 of the playback; therefore, there was no evidence that males changed their song-switching rates (χ^2^_1_ = 0.30, *N* = 12, *P* = 0.58; effect size = 0.02 ± 0.04; Fig. 3A). Females had a song-type switching rate of 24.11 ± 6.70 changes per hour before the playback (day 0) and 14.05 ± 4.50 changes on day 3 of the playback; this provides weak evidence that females have lower song-type switching rates in response to a playback-simulated increase in local population density (χ^2^_1_ = 2.91, *N* = 12, *P* = 0.09; effect size = 0.10 ± 0.06; Fig. 3B).

Anecdotally, we observed changes in male movement behavior following the playback manipulation. After 3 d of the experiment, males tended to sing from higher song perches, reaching the basal branches of high trees (more than 12 m in height). Females tended to remain in areas closer to the nest during the experiment, and when singing, were located in perches at low to moderate heights (1 to 5 m height).

## Discussion

Rufous-and-white wren males altered their vocal behaviors after 3 d of playback simulating an increase in the number of conspecific neighbors. Females did not show evidence of altering their vocal behaviors, although there was high variation in the vocal response among females. Male song plays a major role in the early stages of territory defense, particularly in light of increased local density, and female songs and female duets may serve a different function, more associated with territory maintenance. Playback loudspeakers were positioned well beyond the territory boundaries of the focal pair, and in response to broadcast male solos, female solos, and duets at natural rates and sound levels, we did not observe aggressive responses from the playback subjects. Yet, we did find acoustic changes to the subjects’ behavior in response to the songs heard beyond their territory boundaries, where the independent songs of males increased 3 d after the apparent change in local conspecific density, as well as other smaller changes in behavior, including a marginal tendency of decrease in female song-switching rate. Overall, these results are consistent with the idea that the vocal behavior of wrens varies with social information gathered from beyond territory boundaries, responding to apparent changes in the local density of conspecific animals. We conclude that birds are sensitive to acoustic cues of conspecific density arising from sounds beyond their territory boundaries and that they use this public information to guide their signaling behavior.

The independent song rate of male rufous-and-white wrens increased with the playback-simulated change in local density, supporting our hypothesis that vocal behavior varies due to competition for resources. As the number of pairs in the subjects’ neighborhood increases, the same resources are in higher demand through competition at territory boundaries, and more signaling is required to defend established territories. Furthermore, more neighbors may increase the possibility of future territorial takeovers. There was no evidence of a change in song-type switching by males. Changes in male song rate as a response to changes in the density of conspecific birds have been observed in a wide range of species in the temperate zone, including chipping sparrow, *Spizella passerina* (Liu 2004), eastern kingbird, *Tyrannus tyrannus* (Sexton et al. 2007), dusky flycatcher, *Empidonax oberholseri* (Stehelin and Lein 2014), as well as one tropical bird, the white-browed sparrow-weaver, *Plocepasser mahali* (Voigt et al. 2021). Males tend to increase their song rate with an increase in the number of conspecific neighbors (Sánchez and Mennill 2024).

Females’ independent song rate was slightly higher following the playback manipulation. Song-type switching was somewhat less frequent when the apparent local density of conspecific birds was increased. In a previous study on the same population of rufous-and-white wrens using a 17-yr archive of passive recordings to test the effect of conspecific neighbors on singing behaviors, females switched song types more often when they had one territorial neighbor compared with 2 territorial neighbors, although they showed no difference when they had 0 or 3 territorial neighbors (Owen and Mennill 2021). Taken together, these results suggest that the female song-switching rate could be influenced by the density of conspecific neighbors, although further studies are needed to confirm this pattern. Female rufous-and-white wren independent song rate in natural conditions is quite low (Mennill and Vehrencamp 2005), even during the periods with the highest peaks of song output for this species (Topp and Mennill 2008), and therefore the detection of small changes in female song rate merits attention. Birds in our population typically have 0, 1, 2, and, rarely, 3 or more neighboring pairs (Owen and Mennill 2021). Our playback manipulation, where we simulated 3 neighbors in addition to existing neighbors, resulted in an environment with a high local density of conspecific neighbors. Females that responded with high song type switching may have perceived this as an environment with more competition for territorial resources, or an environment when more extra-pair copulations are likely to occur (extra-pair copulations occur between neighbors in this species; Douglas et al. 2012) and adjusted their song rate and song type switching rate to communicate with the simulated neighbors. Reduction in song type switching rate has been associated with agonistic responses in other species (Deoniziak and Osiejuk 2020). Extra-pair copulations occur at relatively low levels in rufous-and-white wrens, although still at sufficient levels to influence realized reproductive success (Douglas et al. 2012); consequently, an opportunity for extra-pair copulations is one possible explanation for females’ vocal responses to a higher density of neighbors (i.e. similar independent song rate, similar contributions to duets, and slightly lower song-type switching for females). Lastly, the heterogeneity of the female’s vocal responses could also be influenced by the strength of their pair bond with the male and the pair’s reproductive status.

Duet data showed little or no evidence of response to the playback-simulated increase in local density, although the duet rate was higher, on average, on the third day of the playback experiment compared with the preplayback levels. Previous work in this population of rufous-and-white wrens showed that duets are important in territorial interactions (Mennill 2006; Mennill and Vehrencamp 2008; Hick et al. 2016), and for this reason, we expected that duet rate would increase when new territorial neighbors were simulated. Based on the territorial defense function of duets for this species, there are potential explanations for our lack of observed changes in duet rate following the multispeaker density manipulation. First, the number of duets per hour by our simulated neighboring pairs, outside of the context of a territorial intrusion, is quite low. In natural conditions, the duet rate reaches its highest peak in April and May, during the prebreeding, nest building, and fertile periods, when duets represent 13.8% ± 2.7 of all recorded vocalizations (Topp and Mennill 2008). In our study, the duet rate of all the experimental subjects, also recorded between April and May, showed an average duet rate of 6.5 ± 1.6 duets per hour. Therefore, there is only a small opportunity to detect changes in duet rates. Second, the changes in local density that we simulated in this experiment occurred beyond the territorial boundaries of the subjects, with a relatively low level of duetting behavior from the simulated neighbors; this may not have been perceived as a sufficiently strong stimulus to influence the duet rate of the subjects. Indeed, when previous investigations involved playback of a duetting pair inside a territory boundary, with a high duet rate from the simulated intruders (as in Mennill 2006; Mennill and Vehrencamp 2008; Hick et al. 2016), territorial birds increase their duet rate dramatically.

Our use of an array of 6 loudspeakers to increase the apparent density of neighboring pairs is the first such acoustic manipulation designed to explore the effects of the density of conspecific birds (Sánchez and Mennill 2024). With extensive background research on our study population, we were able to simulate new, unfamiliar territorial neighbors that sang at realistic rates of independent songs for males and females, and realistic rates of duets (Mennill and Vehrencamp 2005; Topp and Mennill 2008). The song repertoire of our simulated pairs consisted only of 3 song types for each male and female in each of the 3 simulated neighboring pairs. When sampled extensively (Harris et al. 2016), both sexes have larger repertoires than this (average male repertoire size: 10.8; average female repertoire size 8.5). Variation in song repertoire size is a valuable additional component that might be considered in future experiments, holding the song rate constant but varying the repertoire size of simulated pairs. Additional important considerations are related to the location of the speakers; we were extremely cautious in keeping the simulated pairs beyond the territory boundaries, in order to avoid simulating intruders to the area where the subjects had been observed defending their territory, with a minimum distance from the nests set at 90 m. Importantly, our experimental design allows us to manipulate apparent density without more severe influences on bird populations associated with experimental removals (Shutler and Weatherhead 1991; Liu 2004; Sillett et al. 2004) or natural removals (Stehelin and Lein 2014). Given that all of our playback-simulated neighbors were unfamiliar to the playback subjects, it is noteworthy that the density playback was accompanied by quite a novel manipulation from the perspective of the subjects, with the arrival of 3 unfamiliar pairs beyond their territory boundaries.

In conclusion, we found support for the idea that the density of conspecifics has an effect on rufous-and-white wren males’ vocal behavior. Although the difference was small for females, their decline in song-switching rate is still intriguing. We have long recognized that nearby animals modify the social context of communication (Naguib 2005; Snijders and Naguib 2017), and when the density of conspecific neighbors is high, more signaling may be required and more complex forms of singing behavior may facilitate communication with a broader audience. Based on the findings from the density manipulation experiment we performed here, males appeared to perceive an increased density of neighbors as potential rivals, which induced an increase in song rate, whereas females showed high variation in the response to an apparent increase in the number of neighbors, appeared to perceive the new neighbors as either extra-pair copulation opportunities or potential rivals. Given that global anthropogenic effects are diminishing population densities in wild animal populations worldwide, our results suggest that territorial animals may become quieter as population densities decline, especially if more neighbors induce higher song output for territorial songbirds, highlighting a heretofore undescribed dampening effect of human influence on natural soundscapes.

## Supplementary Material

arae075_suppl_Supplementary_Figure

## Data Availability

Analyses reported in this article can be reproduced using the data provided by Sánchez et al. (2024).
