# Supplementary figures and images for: Neighbors affect vocal behavior of tropical wrens: a multispeaker density-manipulation experiment
Source: Behav Ecol. 2024 Sep 17;35(6):arae075. doi: 10.1093/beheco/arae075 (PMC11461926; doi:10.1093/beheco/arae075)

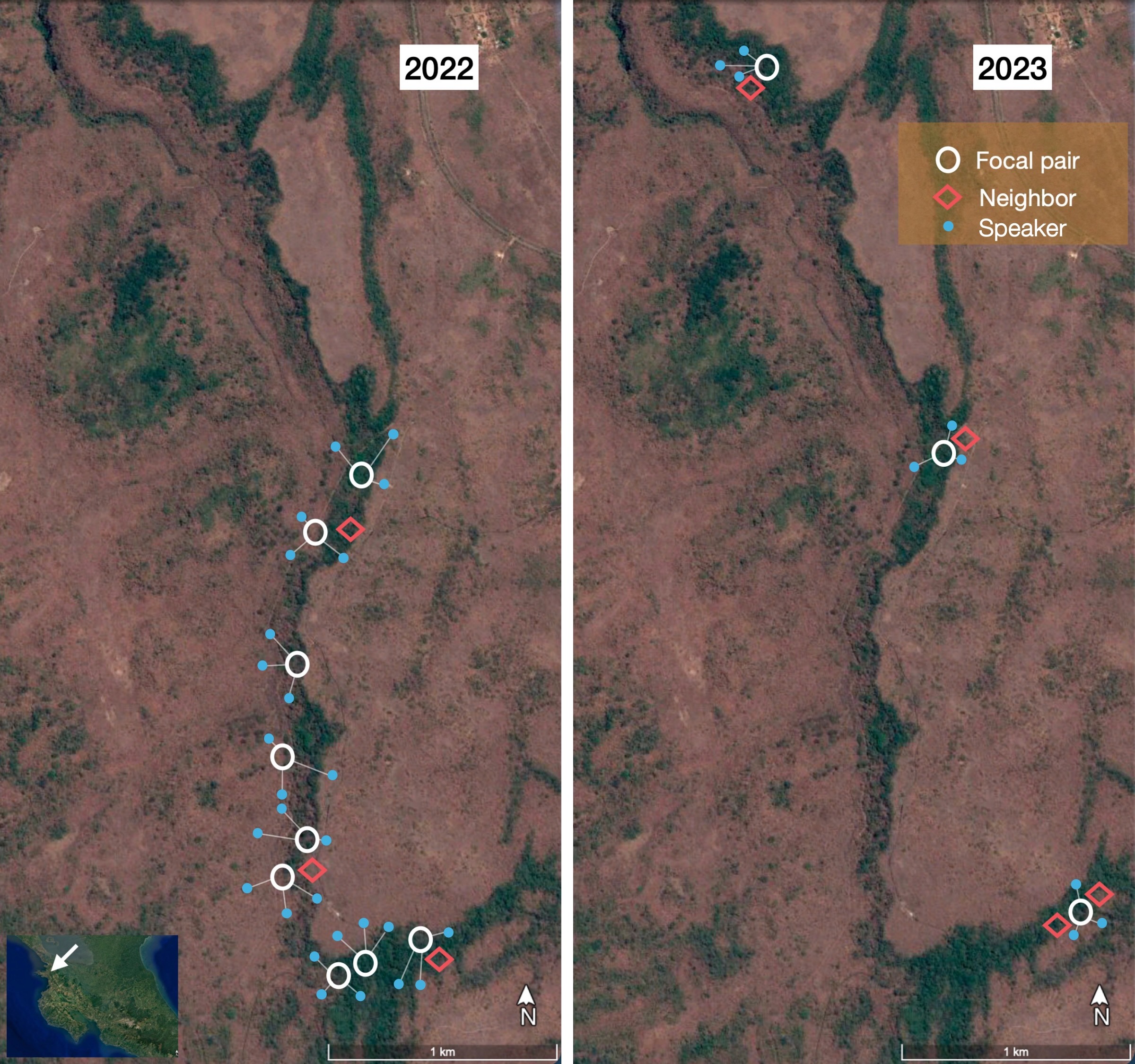

Supplement: arae075_suppl_Supplementary_Figure [file arae075_suppl_supplementary_figure.jpeg]
